# Clinical Benefit of Pegylated Liposomal Doxorubicin and High Prevalence of Pre-Existing Psychiatric Conditions in Patients with Desmoid-Type Fibromatosis

**DOI:** 10.3390/cancers17020293

**Published:** 2025-01-17

**Authors:** Andrea Espejo Freire, Keith M. Skubitz

**Affiliations:** Department of Medicine, The Masonic Cancer Center, The University of Minnesota Medical School, University of Minnesota, Minneapolis, MN 55455, USA; espej018@umn.edu

**Keywords:** desmoid-type fibromatosis, aggressive fibromatosis, desmoid tumor, pegylated liposomal doxorubicin, psychiatric comorbidities, polycystic ovary syndrome

## Abstract

**Background/Objectives:** Desmoid-type fibromatosis (DTF) is a locally invasive tumor composed of myofibroblast-like cells and collagen; it does not metastasize but can cause significant local morbidity. Most sporadic cases are associated with mutations in the CTNNB1 gene, which encodes beta-catenin. Various treatments have been used with differing efficacy and toxicity profiles. At our institution, pegylated liposomal doxorubicin (PLD) has become the preferred treatment for patients with DTF. We aim to describe our experience using PLD in patients with DTF who require treatment. **Methods:** A retrospective review of 61 DTF patients (41 females, 20 males) treated between 2000 and 2023 was conducted to assess the efficacy and toxicity of PLD. **Results:** Of the 26 patients treated with PLD, 23 had follow-up clinical data to assess benefit. Twenty-one showed clinical benefit, and only one progressed. Two patients did not benefit from PLD due to infusion reactions and chose alternative therapies. The primary side effect of PLD was hand-foot syndrome (HFS), but dose reduction and extended intervals allowed most patients to tolerate treatment. Other treatments, such as methotrexate, vinblastine/vinorelbine, and sorafenib, also showed activity but had significant toxicities, including severe HFS, malaise, and hypertension. Interestingly, 31 out of 61 patients had a pre-existing history of psychiatric conditions (primarily depression and anxiety), and 6 of 41 women had personal or family history of polycystic ovary syndrome (PCOS). Additionally, 15 patients had obesity, and 4 had hypothyroidism. **Conclusions:** PLD is an effective and well-tolerated treatment for DTF, with good clinical responses at lower, tolerable doses. The association of pre-existing psychiatric diagnoses, PCOS, and obesity warrants further investigation.

## Simple Summary

Desmoid-type fibromatosis (DTF) is a non-metastatic tumor composed of myofibroblasts that can cause local invasion and significant morbidity. Most cases are associated with a mutation in the CTNNB1 gene, which encodes beta-catenin. Various treatments for DTF have been used with differing efficacy and toxicity profiles. At our institution, pegylated liposomal doxorubicin (PLD) is the preferred treatment for DTF patients. A retrospective review of 61 patients (41 women, 19 men) from 2000 to 2023 showed that 26 patients were treated with PLD. Of 23 patients who had follow-up data, 21 had a clinical benefit, and only 1 progressed. Common toxicities included hand-foot syndrome (HFS), which was managed by dose adjustments. Other treatments such as methotrexate and sorafenib were also used in our cohort. Notably, many patients had a history of psychiatric conditions, obesity, or polycystic ovary syndrome (PCOS). Overall, PLD is an effective and well-tolerated treatment for DTF. The high incidence of pre-existing psychiatric disorders warrants further investigation.

## 1. Introduction

Desmoid-type fibromatosis (DTF) is a locally invasive soft tissue tumor that rarely metastasizes and exhibits a highly variable clinical course. It consists of poorly circumscribed myofibroblast-like cells with heterogeneous collagen deposition [1,2,3]. Histologically, DTF demonstrates intra- and inter-tumor heterogeneity, with regions of low cellularity and high collagen deposition alongside more cellular areas with less dense nuclei and higher mitotic activity [3]. Most sporadic DTF cases are associated with mutations in the CTNNB1 gene, which encodes beta-catenin. A smaller proportion of cases are linked to germline mutations in the APC gene, predisposing patients with Familial Adenomatous Polyposis (FAP) to develop DTF; APC is involved in beta-catenin degradation and thus regulates free beta-catenin concentration [1,2].

Traditionally, DTF was managed with surgical resection. However, high recurrence rates and advancements in understanding its biology have led to a shift toward initial surveillance followed by systemic therapies in cases of clinically significant progression [1,2,4,5,6,7,8,9,10,11,12]. Several treatments have been explored for DTF, each with distinct toxicities and varying efficacy. A combination of methotrexate and vinblastine was one of the first chemotherapy treatments reported for DTF with responses in 30–50% of patients [13,14,15,16,17].

Other agents have also been reported to have activity, including nonsteroidal anti-inflammatory drugs, colchicine, and traditional chemotherapies, such as free doxorubicin, pegylated liposomal doxorubicin, oral vinorelbine, methotrexate, and dacarbazine [1,18,19,20,21,22,23,24,25,26,27]. Hormonal manipulation has also been used [1,23]. Historically, intense chemotherapy, particularly combination regimens, has been associated with faster and more pronounced response rates. These regimens were commonly preferred in cases with organ-threatening involvement or severe symptoms. A 2012 study by the French Sarcoma Group, which included 62 patients, found significantly higher response rates with anthracycline-containing regimens (54% vs. 12%) [28]. Similar results were reported in a study of 68 patients from Memorial Sloan Kettering, which also showed the highest response rates with anthracyclines [29]. Notably, the combination of doxorubicin and dacarbazine produced significant responses, including complete remissions in several cases previously considered unresectable. This was particularly true for patients with intra-abdominal DTF associated with FAP. For these reasons, doxorubicin and dacarbazine have often been recommended as first-line treatment in such challenging cases [21,30,31,32,33].

The activity of the tyrosine kinase inhibitor (TKI), imatinib, and later other TKIs, was demonstrated in DTF, and subsequent reports described the activity of other TKIs in imatinib-resistant cases [34,35,36,37,38,39,40]. A phase 3 trial evaluating sorafenib versus placebo found objective responses in 33% of sorafenib-treated patients compared to 20% in the placebo arm. Grade 1–2 toxicities, such as rash, hypertension, diarrhea, and fatigue, were observed in over 50% of patients, with grade 3 adverse events occurring in 30% [41]. More recently, gamma-secretase inhibitors targeting Notch1 signaling pathways, which co-regulate with beta-catenin, have emerged as another potential treatment for DTF. In a phase 3 trial of 142 patients, nirogacestat demonstrated an objective response rate of 44% compared to 8% in the placebo group. However, treatment was associated with significant adverse effects, including diarrhea (84% of patients), nausea, fatigue, and rash. Ovarian dysfunction was seen in up to 75% of women of childbearing age [42].

At our institution, we have gradually adopted pegylated liposomal doxorubicin (PLD) for the treatment of desmoid-type fibromatosis (DTF) when therapy is indicated. Advances in chemistry and nanotechnology have led to the development of nano-sized drug delivery systems aimed at improving pharmacokinetics, enhancing tumor specificity, and overcoming mechanisms of resistance. These technologies are designed to target specific tumor conditions, such as inflammation, hypoxia, and increased blood vessel permeability, which arise from altered tumor vasculature and impaired lymphatic drainage [43].

Pegylation, which involves attaching polyethylene glycol (PEG) fragments to a drug, increases its molecular mass and slows clearance. This modification may reduce antibody binding to the modified particles, decrease toxicity to normal tissues with less permeable capillaries, and ultimately improve the therapeutic index. PLD was the first FDA-approved drug in this class, gaining approval in 1995 for the treatment of Kaposi sarcoma [43]. PLD is a liposomal formulation of doxorubicin, where the drug is encapsulated in liposomes coated with methoxypoly (ethylene glycol) (mPEG). The mPEG coating reduces uptake by the reticuloendothelial system, leading to a much longer half-life in the bloodstream compared to non-pegylated liposomes, and limits exposure to certain organs, such as the myocardium [44]. Additionally, studies have shown that PLD can increase drug delivery to some tumors [44], and, in at least one case, has led to a long-term cure of sarcoma after the failure of free doxorubicin treatment [45].

While liposomal formulations provide therapeutic advantages for certain drugs, they may also be associated with unexpected toxicities. For example, PLD has a highly favorable efficacy–toxicity ratio compared to free doxorubicin, requiring no antiemetics and causing minimal myelosuppression, alopecia, and cardiotoxicity. However, PLD is associated with a low incidence of infusion reactions, which appears to be related to the liposomal formulation itself [46]. The most common limiting toxicity is hand-foot syndrome. This favorable toxicity–efficacy profile allows for prolonged treatment with PLD, without the limitations of cardiotoxicity or myelosuppression that are seen with free doxorubicin. Data suggest that PLD achieves objective response rates ranging from 30% to 50%, with some complete responses and disease control in over 70% of patients [18,47,48]. This study aimed to evaluate the relative efficacy and toxicity of PLD in our patient population with DTF and to describe the incidence of important medical comorbidities in patients living with DTF.

## 2. Materials and Methods

After institutional board approval, we conducted a retrospective chart analysis of patients in our electronic medical record with a diagnosis of DTF treated by one clinician (KMS) in our division between 2000 and 2023. Mutation status was annotated when available. Collected data included age at diagnosis, sex, history of APC, location of the tumor, mutation status, and treatment modalities with associated toxicities. Due to the retrospective nature of the study and the known limitations of imaging in DTF, we used the operational definition of “clinical benefit” defined by the treating physician. Clinical benefit was inferred based on several criteria, including documented size reduction, decrease in tumor enhancement, stability of size following prior progression, symptom improvement, and ongoing treatment continuation. Additionally, we collected data on treatment-related toxicity, the need for re-treatment, and relevant comorbidities.

## 3. Results

We identified 61 patients with DTF treated at the University of Minnesota from 2000 until 2023 by our group. In some cases, patients were treated by multiple providers at different institutions. Demographic characteristics, the mutation status, and the location of DTF are summarized in Table 1.

In our cohort, 33 patients had genetic testing to identify the driving genetic mutation. Twenty-nine patients had a mutation in CTNNB1: T41A mutation in 15 patients, S45F mutation in 8 patients and S45P in 6 patients. Four patients had a mutation in APC.

Treatments used as first or second line included surgical resection in 6 patients (9.8%), PLD in 26 patients (42.6%), sorafenib in 9 patients (14.7%), other chemotherapies in 13 patients (21.3%), non-steroidal anti-inflammatory drugs (NSAIDs) in 3 patients (4.9%) and 2 patients underwent cryoablation (3.3%). No treatment was necessary for 19 patients (31.1%) (Table 2).

In the patients treated here, the PLD dose was frequently reduced, or the treatment interval increased, to maintain an acceptable quality of life primarily related to hand-foot syndrome (HFS). The starting dose was typically 45 mg/m^2^ every 28 days in patients in whom an early response was desired. In patients where there was concern about potential toxicity, the initial dose used was 40 mg/m^2^ every 28 days or 20 mg/m^2^ every 2 weeks. While cooling of the hands, feet, or mouth was not used with the first cycle, cooling of potentially affected sites before and during infusion and intermittently for the next two days was often used for later cycles to minimize HFS toxicity. In many cases dose reduction was necessary; importantly, this reduced dose was still effective. Pre-medications were not used unless there was an infusion reaction. Most patients who experienced an initial infusion reaction could subsequently receive PLD after symptoms resolved. The most common significant infusion reaction, typically manifested by back pain or shortness of breath, did not appear to be IgE mediated; PLD in vitro causes neutrophil adhesion to human umbilical vein endothelial cells in serum-free medium [46].

### 3.1. Clinical Benefit

Of 23 patients treated with PLD with available follow-up data, 21 were felt to derive clinical benefit (91.3%) and only 1 eventually progressed on PLD. The two patients who did not benefit from PLD experienced infusion reactions to PLD and opted to pursue alternative therapies.

After treatment with PLD, 9/21 patients required subsequent treatments at various time points: 5 received sorafenib, 1 underwent cryoablation, and 3 were re-treated with PLD and had clinical benefit. Table 2 shows therapies used as first line and second line.

### 3.2. Toxicity Profile

The side effect profile aligned with what is reported in the literature. Two patients receiving PLD experienced an infusion reaction and preferred alternative treatment. The main toxicity of PLD was HFS but dose reduction and lengthening intervals usually allowed a tolerable regimen. Only patients with infusion reactions moved to receive a different regimen due to toxicities.

Of 16 patients who received a starting dose of 45 mg/m^2^ every 28 days, 11/16 had either a dose reduction or delayed treatment due to HFS and received a median of 10.5 cycles (range 3–13). All three patients who received a starting dose of 40 mg/m^2^ received a dose reduction and received 7, 10, and 18 cycles. All four patients who received a starting dose of 20 mg/m^2^ every 2 weeks had a change in dose or interval and were treated for 10, 16, 18, and 29 months.

Methotrexate and vinblastine/vinorelbine were difficult to tolerate for patients but had clear activity. Sorafenib also had significant toxicity including HFS and hypertension; hypertension did not always resolve after stopping sorafenib. 

### 3.3. Coexisting Medical Conditions

Surprisingly, we observed that (50.8%) (31/61) patients had documented significant pre-existing psychiatric disease, usually depression/anxiety. Of note, the incidence of psychiatric conditions varied based on mutation status. Among patients with the S45F CTNNB1 mutation, 62% had a psychiatric condition (3/6 women and 2/2 men). In the group with the S45F CTNNB1 mutation, 50% of women (3/6) had a psychiatric condition. Among patients with the T41A CTNNB1 mutation, 20% had a psychiatric condition (2/9 women and 1/6 men). Finally, 50% (2/4) of male patients with APC mutations had a psychiatric condition.

In addition, 9.7% (4/41) of women had polycystic ovary syndrome (PCOS); 2 women without documented PCOS had a mother or sister with PCOS. Additionally, 24.6% (15/61) of patients had significant obesity. Hypothyroidism was present in 8% (5/61) of patients. Table 3 summarizes the co-occurring medical conditions.

### 3.4. Unusual Desmoid-Type Fibromatosis Cases

Five cases included in this report have been previously reported: one as a case of imatinib-resistant, sunitinib-responsive DTF [38], two as cases of DTF that tracked along nerves appearing as multifocal DTF despite being a single contiguous lesion [49], one as a case of unusual multifocal recurrence [49], and one as a DTF in which CTNNB1 analysis suggested a large proportion of the DTF appearing cells in the tumor were derived from myofibroblasts with no CTNNB1 mutation [50]. One case that did not recur after surgery was previously reported as a case of DTF with no mutation in CTNNB1 or APC but with mutations in RAD51C and MYST3 [51].

## 4. Discussion

This study found a high rate of symptom control of DTF with doses of PLD that were tolerable. The dose of PLD used here is noteworthy. While the early development of chemotherapy often focused on the maximally tolerated dose, our results found good tumor control at comparatively low doses of PLD. The use of pegylated liposomal doxorubicin (PLD) is supported by its favorable toxicity profile, making it an attractive treatment option for desmoid-type fibromatosis (DTF). The pegylation of PLD reduces its uptake by the reticuloendothelial system, leading to a longer half-life and a significant reduction in the toxicities typically seen with free anthracyclines [44,52,53]. The primary limiting toxicity observed with PLD in this cohort was hand-foot syndrome (HFS), which was related to dosing and scheduling. While dose reductions and/or changes in treatment interval were common in the current study, the toxicity profile of PLD was generally manageable, and dose reductions did not appear to detrimentally affect treatment efficacy.

Notably, PLD has not been associated with significant cardiac toxicity, even at cumulative doses of 1500–3500 mg/m^2^, as seen in ovarian cancer and sarcoma treatment [52,53,54,55]. Consequently, routine cardiac surveillance is not recommended unless there is a pre-existing clinical risk [56,57]. Several reports have described the utility of PLD treatment for DTF in both adults and children [18,58,59]. A recent retrospective study from three US sarcoma centers compared the efficacy of sorafenib and anthracyclines in DTF treatment. While anthracyclines showed higher response rates (ORR 37% vs. 13%) and superior 1-year progression-free survival (PFS) (73% vs. 59%), they also had more severe adverse effects. Notably, only 20% of these patients received PLD, with the rest receiving free doxorubicin, highlighting the advantages of PLD [60].

Various TKIs have been reported to have activity in DTF, including pazopanib, which has been reported to have improved efficacy and an improved toxicity profile relative to methotrexate-containing regimens [61]. In an open-label phase II trial (DESMOPAZ), 72 patients with progressive desmoid tumors were randomly assigned to pazopanib at 800 mg daily or to chemotherapy with intravenous methotrexate (30 mg/m^2^) plus vinblastine (5 mg/m^2^) given weekly for 6 months, then every 2 weeks for another 6 months. After a median follow-up of approximately 23 months, pazopanib increased the proportion of patients who had not progressed at 6 months relative to chemotherapy (84% vs. 45%), with a nonsignificant trend towards improved response rates [61]. Nevertheless, grade 3 or higher treatment-related adverse events included hypertension and diarrhea with pazopanib, requiring dose modification for toxicity.

Two placebo-controlled trials in DTF have demonstrated the benefit of chemotherapy. In one study of sorafenib, 400 mg orally daily found a response rate of 16/49 (33%) in the sorafenib arm versus 7/35 (20%) in the placebo group, with an estimated 2-year progression-free survival (PFS) of 81% (95% CI 69–96) with sorafenib vs. 36% (95% CI 22–57) in the placebo arm [41]. A trial comparing the gamma-secretase inhibitor, nirogacestat, with placebo in 142 patients with progressive DTF showed an improvement in PFS, with the likelihood of being event-free at 2 years at 76% (95% CI, 73% to 92%) with nirogacestat and 44% (95% CI, 40% to 64%) with placebo and an objective response rate of 29/70 (41%) in the nirogacestat group versus 6/72 (8%) in the placebo arm [42]. Frequent adverse events with nirogacestat included diarrhea in 84% of patients, nausea in 54%, fatigue in 51%, ovarian dysfunction in 75% of women of childbearing potential, and ~20% of patients required dose reduction [41]. Notably, despite the entry criterion of progressive disease in the 12 months before entering the trial, ~44% of patients treated with placebo had stable disease at 24 months and ~20% had an objective response. Interestingly, in patients who had no previous treatment, there was no difference in PFS between the nirogacestat and placebo groups. The response rates and stable disease rates in the placebo arms of the two randomized trials are noteworthy given that the entry criteria required disease progression [43].

Sorafenib and nirogacestat have shown activity in DTF in placebo-controlled randomized trials but still carry a high risk of toxicity, with side effects affecting 50–80% of patients in phase 3 trials [41,42]. While nirogacestat is the only drug formally approved for the treatment of DTF in the US, no study has compared the effects of known different active agents. Recent studies also highlight the efficacy and tolerability of oral vinorelbine as an emerging treatment option for DTF [19,20]. While our study has the limitation of a retrospective unplanned data review with the usual caveats, our results and other reports suggest PLD as another attractive first-line alternative.

Despite recent advancements, the optimal first-line treatment for DTF, the ideal duration of therapy, and whether treatment should be stopped abruptly or tapered remain unclear. Recently, a retrospective study looking into treatment duration with sorafenib found that patients who received over one year of treatment were associated with a lower need for subsequent therapies after discontinuation [60].

A large multi-institutional study of 259 DTF patients found that mutations in CTNNB1 or APC did not significantly affect the response to systemic therapies. However, a worse prognosis was observed in patients with APC mutations, CTNNB1 wild-type, or other mutations [62]. There is evidence that normal fibroblasts/myofibroblasts are recruited to the tumor to varying degrees by the release of factors from mutant DTF cells that may recruit normal myofibroblast precursors from a precursor pool [1,3,63,64]. PTX3 has recently been described as one factor produced by stromal cells that can increase the proliferation of mutant myofibroblasts in DTF [64]. The relationship between treatment response and tumor biology, particularly mutation status and degree of tumor infiltration by nonmutated myofibroblasts, requires further exploration.

The high incidence of pre-existing psychiatric conditions (approximately 50% of patients in our cohort) is noteworthy. A study of 94 DTF patients also found high rates of anxiety, depression, and poor well-being, with 64% of patients reporting moderate to severe distress [65]. Our findings align with this, emphasizing the importance of addressing these comorbidities in the management of DTF. Although the numbers in this study are small, the distribution of DTF mutations may differ depending on the presence of pre-existing psychiatric conditions. Given the complex interplay between the brain, neurohormonal systems, and immune function, these psychiatric conditions may reflect underlying neuroimmune abnormalities that could influence DTF biology [66]. This area warrants further investigation.

Additionally, the high incidence of polycystic ovary syndrome (PCOS) in our cohort is intriguing, and its potential association with DTF warrants further investigation. Further studies exploring the role of neurological disorders and PCOS in DTF development are needed to better understand these complex relationships.

## 5. Conclusions

The optimal first-line chemotherapy for DTF is unclear. PLD is an attractive treatment option for those patients with DTF requiring treatment. Good responses were observed at well-tolerated PLD doses, which were often much lower than the initial dose. It is not necessary to use poorly tolerated PLD doses for DTF. Unresolved questions include the optimal duration of treatment and whether the degree of tumor infiltration by normal non-mutant myofibroblasts affects the outcome and response to treatment. The inter-individual heterogeneity of tumor biology complicates such studies. Pre-existing psychiatric conditions established well before diagnosis of DTF were present in ~50% of our cohort and metabolic syndrome-related conditions were present in ~45%. A surprising incidence of PCOS was also observed.

## Figures and Tables

**Table 1:** Demographic characteristics of patients with DTF treated at the University of Minnesota from 2000 to 2023.

Variable	*N*	Median (Range)
**Age in years**	61	37 (19–72)
**Gender**	***N***	**Percent%**
Male	20	33
Female	41	67
**Mutation Status**	***N***	**Percent%**
CTNNB1	29	47.5
APC	4	6.5
Unknown	28	46
**Location**	***N***	**Percent%**
Intra-abdominal	17	27.9
Extremities	17	27.9
Abdominal/lumbar wall	11	18
Thoracic	7	11.5
Head and Neck	5	8.2
Pelvis	4	6.6

**Table 2:** Treatment modalities for desmoid-type fibromatosis.

**Treatment First-Line**	***N* (61)**	**Percentage**
No treatment	19	31.1%
PLD	19	31.1%
Surgery	6	9.8%
Sorafenib	4	6.5%
Methotrexate/Vinblastine	6	9.8%
NSAIDs	3	4.9
Methotrexate/Vinorelbine	1	1.6%
**Treatment Second line**	***N* (14)**	**Percentage**
PLD	7	50%
Re-treatment with PLD	1	7.1%
Sorafenib	5	35.7%
Cryoablation	1	7.1%

**Table 3:** Comorbidities in patients with desmoid-type fibromatosis.

Comorbidities	*N*	Percentage
Pre-existing psychiatric disease	31	50.8%
Obesity	15	24.6%
PCOS	4	9.7%
Family Hx of PCOS	2	4.9%
Hypothyroidism	4	6.5%

## Data Availability

The raw data supporting the conclusions of this article will be made available by the authors upon request.
